# Validation of a community-based application of the Portuguese version of the survey on Social and Emotional Skills – Child/Youth Form

**DOI:** 10.3389/fpsyg.2023.1214032

**Published:** 2023-08-21

**Authors:** Catarina Castro, Clara Barata, Joana Alexandre, Carla Colaço

**Affiliations:** ^1^Department of Psychology, Center for Psychological Research and Social Intervention – CIS-IUL, ISCTE – University Institute of Lisbon, Lisbon, Portugal; ^2^Institute of Interdisciplinary Research, Center of Interdisciplinary Studies (CEIS20), University of Coimbra, Coimbra, Portugal

**Keywords:** adolescents, children, measures, social and emotional skills, social emotional learning, validation

## Abstract

**Introduction:**

Children and adolescents’ social and emotional skills have been gaining attention in diverse settings. With over 100 conceptual frameworks available, there is now a common move toward framing these skills as social and emotional learning (SEL), assuming that they are not only amiable to development, but also malleable to change as a product of intervention. As such, there is a strong need for a comprehensive measure to effectively evaluate such skills, validated for different age groups in children and young people, and applicable to both educational contexts and community settings.

**Methods:**

This paper presents the validation of the Portuguese adaptation of the Child/Youth form of the Survey on Social and Emotional Skills (SSES), in the scope of the Gulbenkian Academies for Knowledge initiative with a sample of 7,831 participants between 8 and 17 years old (*M* = 11.79, *SD* = 2.94).

**Results:**

Results show that the measure has good internal consistency and sensitivity, while also being sensitive to change over time. Preliminary factor analysis shows promise, although further research is necessary.

**Discussion:**

Discussion reflects on the value of the Child/Youth form of the SSES as a comprehensive measure to be used by community and educational professionals to monitor skill development and improve their work on SEL.

## Introduction

Social and emotional skills are a multidimensional construct that encompasses a set of intrapersonal competencies, important for the overall functioning of individuals, and interpersonal ones, essential for successfully interacting with others (Domitrovich et al., 2017).

Social–emotional learning (commonly referred to as SEL) is the process by which social and emotional skills are developed. According to Weissberg et al. (2015), it is through this process that knowledge, attitudes, and abilities are acquired, which are fundamental to managing emotions, achieving a set of goals, feeling and showing empathy for others, establishing and maintaining interpersonal relationships, and making responsible decisions.

Children and adolescents’ social and emotional skills have been gaining attention in diverse settings, including educational contexts and community settings. The importance of these skills is now also changing policy. For example, these skills have recently been included in the educational reference that guides education policies in Portugal – the Profile of Students Leaving Compulsory School (Martins et al., 2017).

The need for reliable and valid measurements of social and emotional skills is of crucial importance for evaluating SEL interventions. As Duckworth and Yeager (2015) state, measurement matters for many reasons: from a practical standpoint, changing certain competencies is easier when we can measure them, so data is important to inform action. Measurement also helps to inform progress monitoring, and to effectively evaluate SEL programs (Brush et al., 2022). For instance, the *Data Wise* model (Boudett et al., 2013) is an eight-step model that guides educational teams to improve teaching and learning by performing evidence-based analyses, motivating them to apply and systematize practices with the aim of articulating intervention and evaluation prior to implementation, and restructuring the intervention after its conclusion, based on collected data (Boudett et al., 2013).

However, there is great variability in available measures for social and emotional skill assessment, particularly in terms of the behavior they capture, the uniqueness of its constructs (as opposed to some degree of theoretical overlapping), in how comprehensive they are (measuring one single skill versus several dimensions and skills), the respondents they engage with (children, youth, parents, teachers) and format of response (questionnaires, observation measures, tasks; Humphrey et al., 2011). More importantly, SEL assessments tend to “vary greatly depending on the theoretical frameworks that underlie them” (Murano et al., 2021, p. 1).

This variability leads to diverse approaches, and often conceptual confusion, regarding how these skills are defined, how they translate to observable behavior and, consequently, how they are measured, leading most available instruments to be highly specific for the evaluation of a given intervention, or the measurement of a given skill (Martinez-Yarza et al., 2023). In a systematic review by Humphrey et al. (2011), 12 instruments for measuring SEL were found. The authors concluded that many of these measures were not being extensively used and disseminated, and were unevenly distributed across the targeted skills, for example, emotional skills were less evaluated than social skills. The authors also argued that most measures had only been validated for the United States and the United Kingdom, and only for non-diverse groups of children (Humphrey et al., 2011).

More recently, with the increased popularity of social and emotional learning approaches, a systematic review by Martinez-Yarza et al. (2023) identified 25 measures developed over a 20-year period, covering elementary through secondary education, usually targeting some SEL dimensions but not all of them. This review also shows that the most frequently used assessment method was indirect assessment relying mainly on Likert scales, suggesting there is the need for validating brief and user-friendly measurement measures, as well as for using a combination of multi-method and multi-informant assessment to effectively assess these skills (Martinez-Yarza et al., 2023). SEL measures also need to capture the dynamic interaction between individuals and their environment and context (Brush et al., 2022).

### The study on social and emotional skills

Recognizing the central role of SEL at a young age for a healthy and successful adjustment throughout life, the Organization for Economic Cooperation and Development (OECD) developed the Study on Social and Emotional Skills (SSES). The development of the study was underlined by an effort to consolidate and disambiguate knowledge about how SEL skills develop in children and youth, and what aspects of children’s daily settings – family, school, community – promote or hinder this development.

The OECD’s Study on Social and Emotional Skills (and, subsequently, its survey) was framed under the Big Five model, following one of the most common frameworks for personality and skills. However, also following recent trends in the literature, it understands these skills as malleable, learnable and context dependent, as opposed to fixed traits of personality (Kankaraš and Suarez-Alvarez, 2019). The Big Five structure aims to provide a “general outline of how these skills are organized” (Chernyshenko et al., 2018, p. 9), since this five factor structure has been commonly found in personality and skills research in different cultures and settings (e.g., McCrae and Costa, 1997), including for children and young people (e.g., Tackett et al., 2012), and correlates highly with several outcomes throughout life (such as wellbeing, academic and professional success, or physical health; Chernyshenko et al., 2018).

Following a thorough review, the OECD opted for this Big Five structure to guide its approach to social and emotional skills, organizing its study in five dimensions: *Collaboration*, *Task Performance*, *Emotional Regulation*, *Engagement with Others*, *Open-mindedness*. These relate, respectively, with the classic Big Five domains of *Agreeableness*, *Conscientiousness*, *Neuroticism*, *Extraversion*, and *Openness to Experience*. Each dimension then encompasses several individual skills, which are the focus of the SSES.

*Collaboration* is understood as the ability to have sympathy toward others and express altruism, leading to better quality relationships and more pro-social behaviors; it includes the individual skills of *Empathy*, *Trust*, and *Cooperation*. *Task performance* relates to being self-disciplined and persistent, with a tendency to stay on task and to be a high achiever; it includes the individual skills of *Responsibility*, *Self-control*, and *Persistence*. *Emotional regulation* refers to what allows an individual to effectively manage negative emotional experiences and stressors, and it includes the skills of *Stress resistance*, *Optimism*, and *Emotional control*. *Engagement with others* refers to those who are extraverted, energetic, positive, and assertive, having an ease to establish social connections; it includes the skills of *Sociability*, *Assertiveness*, and *Energy*. Lastly, *Open-mindedness* stands as the will to accommodate different perspectives and new experiences, and includes the individual skills of *Curiosity*, *Tolerance* and *Creativity* (OECD, 2021).

As a product of the study, a comprehensive measure was developed, the Survey on Social and Emotional Skills, and was administered to over 60,000 participants of 10 and 15 years of age in 10 cities around the world, collecting data on 15 different social and emotional skills, as well as on sociodemographic, family, school, and community contextual characteristics, with data on students’ skills being reported by students, families, and teachers. SSES was implemented as the first large-scale international survey of SEL (OECD, 2021). Portugal was represented in this study by the Municipality of Sintra, contributing with over 3,000 participants, and thus constituting the sample for the initial Portuguese adaptation of this instrument.

This study, and the resulting survey, stand as a valuable effort to develop a comprehensive measure to the assessment of a broad array of social and emotional skills. The concern for the predictive value of the selected skills, the suitability of a Big Five approach to skills across different cultures and ages, and the comprehensive nature of the questionnaire support its suitability to measure these social and emotional skills, allowing for researchers and practitioners to further delve into the evidence-based promotion and evaluation of SEL.

### Gulbenkian Academies for Knowledge

In 2018, the Portuguese Calouste Gulbenkian Foundation set out to implement a central mechanism for the development and support of innovative solutions for complex societal problems. In order to do so, the Foundation offered to co-fund intervention approaches to SEL, named Gulbenkian Academies for Knowledge (henceforth referred to as Academies), which could include a broad array of domains such as educational, science learning, health, civic participation, among others, under the common umbrella of developing social and emotional competencies of children and youth 0 to 25 years of age across the country. Between 2018 and 2022, the Foundation opened three rounds of applications (2018, 2019, 2020) in order to select 100 community-or school-based projects. Each project could be implemented across 1, 2 or 3 years. Because some Academies chose to test their intervention only in their second year of funding, there were in total 4 cohorts of Academies, across four school years.

To achieve the goal of promoting these skills, 35 Academies chose to implement intervention approaches previously validated using experimental or quasi-experimental evidence, and proven results (such as the *Incredible Years* Program), while the remaining 65 chose to develop and implement pilot approaches, i.e., innovative interventions, designed by each Academy, with the potential to be rigorously evaluated and validated as an effective intervention (*N* = 65). By integrating the GAK initiative, each Academy also committed to the implementation monitoring (Durlak and DuPre, 2008) and the experimental or quasi-experimental impact evaluation of its intervention, with the aim of contributing to the production of knowledge, and the dissemination of evidence-based interventions, without compromising the quality criteria necessary to these processes. All Academies were also recommended to involved at least 100 participants in their impact evaluation, in order to ensure some statistical power in their impact evaluation. Although this was not mandatory, it was strongly recommended, and most of the projects complied to this rule.

In addition to co-funding the intervention, the Foundation offered 100 selected programs the technical support of a Monitoring and Evaluation Team, which assisted Academies in all stages of their evaluation processes, including the development of their Theory of Change, closely monitoring various dimensions of program implementation, and designing experimental, quasi-experimental or descriptive impact evaluation studies, with standardized measures of intervention and control/comparison groups at pre-test and post-test. The M&E Team also provided continuous support and training opportunities to all Academies throughout the initiative. The training model, based on the *Data Wise* model (Boudett et al., 2005), focused on aspects related to monitoring (how to design a Theory of Change, how to observe program implementation, how to use program implementation monitoring data to improve interventions), impact evaluation (how to conceptually align intervention and evaluation, how to select evaluation measures, how to constitute intervention and control groups, how to analyze and discuss results), and ethical aspects inherent to research in the field. The M&E Team did so by providing training sessions, frequent individual consultancy, and visiting the Academies.

Of central importance to this study, is that the Foundation required the use of the SSES as a common metric of impact measurement across Academies. This means Academies were required to use SSES for pre-and post-test assessment of all participants in their evaluation. Because theories of change across Academies varied greatly, and the Foundation wanted to fund intervention approaches with a clear goal, Academies could choose a minimum of two SSES competencies to monitor across evaluation stages. Moreover, no items from the Energy subscale could be used because this skill was not aligned with the theoretical scope of the Foundation work. Academies could complement their evaluation work with other standardized measures of assessment.

### The present study

This study aimed to examine the psychometric properties of the Portuguese version of the Child/Youth Form of the Survey on Social and Emotional Skills (SSES; OECD, 2021) used within the Gulbenkian Academies for Knowledge initiative with a large Portuguese community sample. Specifically, we aimed to: (1) to test the internal consistency of the SSES – Child/Youth Form, by analyzing its internal consistency; (2) to test the scale’s sensitivity to changes in participants’ social and emotional skills between pre-test and post-test; and (3) to explore the factor structure of the SSES – Child/Youth Form.

## Method

### Sample

The study sample included participants from 43 Academies, 5 from validated approaches and 38 from pilot approaches. The requirement to use the SSES as a common impact measure was implemented starting in the second cohort of Academies, because SSES was not available prior. However, due to the low quality and quantity of data from the 2nd edition (2019–2020), which was severely impacted by the outburst of the COVID-19 pandemic midyear, data from these 43 Academies which implemented the SSES Child/Youth Form comes from the third and fourth cohort only (2020–2021 and 2021–2022, respectively). Academies which chose not to administer the SSES in any of its forms, or that only administered its Parent or Teacher Forms, have also been excluded from the present study. Finally, only participants between the ages of 8 and 17 years old were included in this study sample, aiming for testing the validity of this instrument for participants 2 years older and 2 years younger than the participants in both cohorts from the original OECD study (10-and 15-year-old cohorts).

This means inclusion criteria for participants in this study comprised all participants from the two final cohort years of the Gulbenkian Academies for Knowledge initiative between the ages of 8 and 17 years old, with available information on age and sex, as well as with responses on SSES – Child/Youth Form either at pre-test or at post-test. This sample comprises 7,831 participants, 52% of which were female, and with ages ranging from 8 to 17 years old (*M* = 11.79, SD = 2.94). Mean school grade was the 6th grade (*M* = 6.15, SD = 2.85), and the majority of participants (74%) were Portuguese. As for family characteristics, both parents were predominantly Portuguese (85% of mothers and 86% of fathers), and their highest educational level was, on average, high school, although mothers scored higher (mother’s educational level *M* = 3.95, SD = 1.14, father’s educational level *M* = 3.69, SD = 1.21^1^). Most families lived in an urban setting (84%), with a fifth (19%) benefitting from some form of social assistance by social security services (Table 1).

**Table 1:** Participants’ sociodemographic characteristics.

	N	M	SD	Min	Max	Skewness	Kurtosis
Child/Youth							
Age	7,831	11.79	2.94	8	17	0.307	−1.375
School grade	7,676	6.15	2.85	1	13	0.336	−1.309
Child is female	7,831	0.52	0.50	0	1	−0.095	−1.991
Child has special educational needs	4,307	0.07	0.25	0	1	3.418	9.686
Child is Portuguese	7,831	0.74	0.44	0	1	−1.114	−0.760
Child attends a public school	6,677	0.91	0.28	0	1	−2.928	6.574
Child has been held back a school year	5,300	0.14	0.35	0	1	2.058	2.235
Parents							
Mom is Portuguese	4,498	0.57	0.49	0	1	−0.301	−1.910
Mom’s age	5,267	41.61	6.19	18	83	0.003	0.455
Mom’s education	6,110	3.95	1.14	0	6	−0.898	0.321
Mom works	5,349	0.80	0.40	0	1	−1.513	0.290
Mom is married	4,183	0.73	0.44	0	1	−1.056	−0.885
Dad is Portuguese	4,207	0.54	0.50	0	1	−0.149	−1.978
Dad’s age	4,649	44.13	6.89	23	76	0.274	0.652
Dad’s education	5,656	3.69	1.21	0	6	−0.617	−0.373
Dad works	4,872	0.91	0.29	0	1	−2.838	6.056
Dad is married	3,986	0.76	0.42	0	1	−1.245	−0.450
Family							
Family benefits from social assistance	3,509	0.19	0.39	0	1	1.573	0.476
Child has siblings	4,705	0.81	0.40	0	1	−1.551	0.406
Number of siblings	4,705	1.27	1.17	0	27	5.028	77.042
Child lives with at least one parent	5,507	0.95	0.22	0	1	−3.991	13.929
Child lives in an urban setting	3,812	0.84	0.36	0	1	−1.880	1.534

### Measure

The SSES – Child/Youth form (OECD, 2021) is a self-report instrument composed of 120 items, answered in a scale of one (Totally disagree) to five (Totally agree), which allows the assessment of a set of 15 social and emotional skills by child or youth participants aged between eight and 17 years old. It includes the following 15 subscales, with eight items each: Optimism (OPT; “I look at the bright side of life”), Responsibility (RES; “I am a responsible person”), Curiosity (CUR; “I like learning new things”), Self-control (SEL; “I stop to think before acting”), Emotional control (EMO; “I stay calm even in tense situations”), Cooperation (COO; “I get along well with others”), Sociability (SOC; “I make friends easily”), Assertiveness (ASS; “I enjoy leading others”), Creativity (CRE; “I have a good imagination”), Resilience/Stress resistance (STR; “I am relaxed and handle stress well”), Persistence/Perseverance (PER; “I make sure that I finish tasks”), Empathy (EMP; “I know how to comfort others”), Tolerance (TOL; “I like hearing about other cultures and religions”), Trust (TRU; “I believe most people are kind”) and Energy (ENE; “I am full of energy”). The survey could be administered in paper format or online format. Data from the global sample of SSES’s main study by OECD (2021) indicates Cronbach’s alpha’s internal consistency levels between 0.71 (Empathy) and 0.85 (Assertiveness).

### Procedures

#### Data collection

Data was collected directly by each Academy’s team with their participants, having selected the appropriate mechanisms to the specific needs of its setting and sample. However, Academies adopted common data collection and management procedures, as well as ethical procedures, and were closely monitored by the Monitoring and Evaluation team. Therefore, all Academies were required, prior to assessment, to collect informed consent from each participant’s legal tutor, prepare data collection materials (paper versions or online versions of each measure), and prepare adequate locations (e.g., classrooms, community facilities).

Since each Academy would select the SSES subscales that best aligned with their Theory of Change, i.e., that evaluated the social and emotional skills targeted by their intervention, there is great variability in sample size for each subscale. Additionally, regarding pre-test and post-test scores, there is a decrease in sample size across subscales due to missing data: respondents may only have participated in one of the data collection moments, with participant mortality being common at post-test.

Data collection procedures could be managed and implemented by any adequately trained member of the Academy’s team, including teachers, social and youth workers, psychologists, researchers, among others, with supervision. In some instances (particularly with adult participants and/or with the comparison or control groups), the materials were provided, and the participant responded autonomously to the measures. Data was then submitted by the Academies to the M&E Team for cleaning and analysis.

Regarding ethical procedures, aside from the aforementioned written informed consent collected from legal tutors, all Academies were instructed to collect oral assent prior to assessment, and debrief underaged participants of study goals and procedures. Moreover, all data collection and analysis procedures ensured confidentiality, with each participant being granted an ID by their Academy’s team, meaning all data was fully anonymous to members external to the Academy, including the monitoring and evaluation team. The M&E team also granted regular ethics and data protection awareness training sessions to all Academies, and provide countless session of mentoring. All Academies whose data is included in this paper granted their approval for it to be processed and published for this purpose by the M&E Team via signed informed consent.

#### Data analysis

To test the scale’s internal consistency, we calculated Cronbach’s alpha for each subscale and for the overall score. We also observed central tendency measures (i.e., mean), dispersion measures (i.e., standard deviation), and the normality of variables was verified by analyzing asymmetry (skewness) and tailedness (Kurtosis) for each item. To test sensitivity to change over time, we conducted a *t*-test for differences between paired samples to analyze differences in scores between pre-test and post-test at the subscale level and in overall score. Effect sizes and correlations between pre-test and post-test measures were also calculated; Cohen’s *d* measure of standardized mean difference was calculated to attest the effect size on all subscales, whereas correlations between pre-test and post-test aimed to assume that scores from both data collection points positively relate to each other. Finally, a preliminary exploratory factor analysis (EFA) was conducted to explore the factor structure of the SSES – Child/Youth Form. Following (Smith-Donald et al., 2007), we used principal component extraction for the 112-item version of the Child/Youth Form of the SSES, i.e., the original 120-item version, excluding the 8 items from the *Energy* subscale. After confirming the suitability of the data via the Kaiser-Meyer-Olkin (Hutcheson and Sofroniou, 1999) test and Bartlett’s test of sphericity (Dziuban and Shirkey, 1974), a preliminary exploratory factor analysis was conducted with the 112 items to explore the factor structure of the SSES – Child/Youth Form. Resulting components were rotated obliquely using Promax to allow correlation between factors. Cronbach’s alpha was calculated for each emerging construct and provides an index of internal consistency based on the average of the items scores in the construct. We used IBM SPSS, Version 28.0 for the analyses.

## Results

### Internal consistency of the SSES – Portuguese Child/Youth form

Table 2 shows Cronbach’s alpha for each subscale, and correlations between subscales and overall score for the SSES – Portuguese Child/Youth Form at pre-test. Internal consistency levels were overall good, ranging from 0.697 (Empathy) to 0.903 (Persistence/perseverance), while the overall scale showed an excellent level of internal consistency (*ɑ* = 0.951). Moderate to high correlations were found for 12 subscales, ranging between 0.627 (Tolerance) and 0.881 (Persistence/Perseverance). Only two subscales (Assertiveness, *r* = 0.403; and Resilience/Stress Resistance, *r* = 0.480) show low yet significant correlations with the overall scale.

**Table 2:** Cronbach’s alpha, correlations and overall score for SSES – Child/Youth form.

	Number of items	Alpha	Correlation with overall score
Curiosity	8	0.781	0.712**
Responsibility	8	0.856	0.865**
Optimism	8	0.835	0.673**
Emotional control	8	0.725	0.695**
Self-control	8	0.867	0.867**
Assertiveness	8	0.881	0.403**
Cooperation	8	0.783	0.731**
Sociability	8	0.759	0.678**
Creativity	8	0.740	0.629**
Persistence/Perseverance	8	0.903	0.881**
Resilience/Stress resistance	8	0.826	0.480**
Empathy	8	0.697	0.665**
Tolerance	8	0.760	0.627**
Trust	8	0.819	0.651**
SSES Child/Youth Form – overall score	112	0.951	

**p < 0.001.

### Sensitivity of the SSES

#### Descriptive statistics and overall score

Supplementary Table 1 presents descriptive information for the items, subscales, and overall score for the SSES – Child/Youth Form at pre-test.

Overall mean results at pre-test ranged between *M* = 1.96 (SD = 1.09, Resilience Item 3) and *M* = 4.61 (SD = 0.65, Sociability Item 3) at the item level (overall score *M* = 3.49, SD = 0.67), and at the subscale level between *M* = 2.67 (SD = 0.89, Assertiveness) and *M* = 4.15 (SD = 0.53, Cooperation). All items were scored between one and five, with 96 items (85.7% of total) average scoring above the scale’s median. Kurtosis and skewness values for most items presented data skewed to the right and mostly peaked, suggesting a concentration of scores toward the higher end of the scale for most subscales.

#### Sensitivity of the SSES to change over time

Table 3 illustrates differences in SSES – Portuguese Child/Youth Form scores between pre-test and post-test. As shown previously in Supplementary Table 1, overall score at pre-test was *M* = 3.49 (SD = 0.67), with subscale mean scores ranging from *M* = 2.67 (SD = 0.89, Assertiveness) to *M* = 4.15 (SD = 0.53, Cooperation). At post-test, no subscales showed a statistically significant higher mean score than at pre-test, whereas seven subscales showed statistically significant differences in the opposite direction (i.e., with participants scoring higher at pre-test): *Curiosity*, *Responsibility*, *Optimism*, *Self-control*, *Cooperation*, *Sociability*, and *Trust*. This is also true for differences between overall scale scores, with a statistically significant decrease in the score between pre-test and post-test. This indicates that participants self-assessed their social and emotional skills higher (and scoring highly in the 5-point scale) at pre-test, before receiving any intervention. Effect sizes ranged between −0.038 (Tolerance) and 0.290 (Responsibility and Resilience/Stress Resistance) at the subscale level. Correlations between scores at pre-test and post-test were moderate and significant for most subscales, as well as for the overall scale, with correlations ranging from 0.606 (*Emotional Control*) to 0.727 (*Sociability*). Exceptions were found for the subscales *Responsibility* (*r* = 0.440), *Empathy* (*r* = 0.565), *Creativity* (*r* = 0.590), and *Self-control* (*r* = 0.599), although all are statistically significant.

**Table 3:** Differences between pre-test and post-test scores for SSES – Child/Youth form’s subscales.

Subscale	*N*	Mean Diff.	SD	*t*	*df*	*p*	Effect size	Correlation between pre-test and post-test
Curiosity	1,837	0.049	0.505	4.164	1,836	0.000	0.097	0.649**
Responsibility	1,533	0.205	0.706	11.373	1,532	0.000	0.290	0.440**
Optimism	1,744	0.135	0.621	9.070	1,743	0.000	0.217	0.635**
Emotional control	1,796	−0.024	0.659	−1.526	1,795	0.127	−0.036	0.606**
Self-control	1,771	0.038	0.603	2.678	1,770	0.007	0.064	0.599**
Assertiveness	2,440	0.014	0.674	0.989	2,439	0.323	0.020	0.723**
Cooperation	3,170	0.038	0.463	4.559	3,169	0.000	0.081	0.642**
Sociability	2,718	0.086	0.507	8.795	2,717	0.000	0.169	0.727**
Creativity	1,862	0.005	0.582	0.395	1,861	0.693	0.009	0.590**
Persistence/Perseverance	1,590	−0.003	0.572	−0.211	1,589	0.833	−0.025	0.624**
Resilience/Stress resistance	1,591	−0.018	0.636	−1.113	1,590	0.266	0.290	0.704**
Empathy	3,766	−0.005	0.540	−0.561	3,765	0.575	−0.009	0.565**
Tolerance	2,585	−0.021	0.540	−1.936	2,584	0.053	−0.038	0.621**
Trust	2,731	0.078	0.582	6.985	2,730	0.000	0.134	0.689**
Overall score	4,853	0.026	0.400	4.498	4,852	0.000	0.065	0.672**

Negative mean differences indicate the score is higher at post-test. **p < 0.001.

### Validity of the SSES

#### Factor structure of the SSES – Child/Youth form

Initial Kaiser-Meyer-Olkin test (KMO = 0.855) and Bartlett sphericity test (Bartlett, χ^2^ (6216) = 21,893,901, *p* < 0.001) confirmed the adequacy of data to perform a factor analysis (Pasquili, 1999; Tabachnick and Fidell, 2007). Initial confirmatory factor analysis on the 112 items of the SSES – Child/Youth form indicated 25 components with eigenvalue >1 (Kaiser rule), accounting for 69.43% of variance explained. However, not only was this structure difficult to interpret given the underlying theoretical framework, but also most of the components’ variance weights were too low (e.g., <2%). Since we were using data pertaining to only 14 of the 15 original subscales, we chose not to force the extraction of a fixed number of factors drawn from the original instrument. Thus, we then forced the extraction of 10 components – due to it cumulatively accounting for over 50% of total variance explained (e.g.: Marôco, 2018). In the newly obtained factorial structure, one item (*Curiosity* – item 6) did not load onto any component (with a value over 0.32; Tabachnick and Fidell, 2007). For items loading in more than one component, we opted to maintain them where the load was higher, as long as the difference in scores was above 0.2 (Pereira and Patrício, 2008). However, that difference was inferior to 0.2 in 14 items, which led to their exclusion. Excluded items belong to the subscales *Cooperation* (items 2 and 4), *Creativity* (item 6), *Emotional control* (items 2 and 6), *Empathy* (items 3 and 6), *Optimism* (item 1), *Responsibility* (item 5), *Self-control* (item 8), and Sociability (items 2, 3, 5 and 6).Factor analysis was then redone under the same rules, including a final version of 98 items loading into 10 components. This final structure explained 52.38% of total variance, and 69.1% of items presented good to excellent loads (i.e., >0.5; Comrey and Lee, 1992). Four items (*Curiosity* – item 4, *Cooperation* – item 3, *Sociability* – item 4, and *Empathy* – item 5) did not load onto any factor. Table 4 summarily presents the final structure of 10 components, and the corresponding load of the 94 items to its main component, whereas Supplementary Table 2 provides the detailed results of this analysis, with all items’ loads to all components. The final 10 components were named as follows: Component 1 – “Perseverance and Responsibility,” since it includes being persistent, responsible and able to control task related behavior (with items pertaining to the original *Persistence/Perseverance* – 8 items, and *Responsibility* – 5 items subscales); Component 2 – “Curiosity and Tolerance toward diversity” (since it includes all 8 items from the original *Tolerance* subscale, and 2 from *Curiosity*), relating to openness to different contexts and people; Component 3 – “Relations with others,” which includes mostly items from the *Cooperation* (5 items) and *Empathy* (5 items) subscales, as well as one item from *Responsibility* subscales, all relating to one’s ability to collaborate with others, and maintain positive relationships; Component 4 – “Emotional Control and Emotional Resilience,” addressing the ability to manage and control emotions, particularly when facing distressful situations (with items from the original *Emotional control* – 6 items, *Resilience/Stress resistance* – 6 items, and *Self-control* – 1 items subscales); Component 5 – “Assertiveness/Leadership,” which is composed of all 8 items from the original *Assertiveness* subscale; Component 6 – “Trust in others,” which includes all 8 items from the original *Trust* subscale, relating to one’s capacity to believe in other people’s good intentions; Component 7 – “Social optimism,” composed of the remaining 7 items from the original *Optimism* subscale, and three from *Sociability* subscale, relating to one’s positive outlook on life and on starting and maintaining social relations, have friends and an active social life; Component 8 – “Care and concern for learning,” which includes 6 items from the original *Self-control* subscale, 4 from *Curiosity* and two from *Resilience/Stress resistance*, related to one’s eagerness to learn, emotional concern and care in performing tasks; Component 9 – “Creativity – Imagination,” including 3 items from the original *Creativity* subscale and one from *Curiosity*, relating to the ability to fantasize and imagine new scenarios; and Component 10 – “Creativity – New solutions,” which includes 4 items from the original *Creativity* subscale and one from *Responsibility*, related to the ability to come up with new ideas and original solutions.

**Table 4:** Factorial structure for the SSES – Child/Youth form (summarized results).

Components									
1	2	3	4	5	6	7	8	9	10
I leave things unfinished* 0.848	I love to learn about other countries and cultures 0.780	I am reliable and can always be counted on 0.695	I get mad easily* 0.761	I like to be the leader of a group 0.916	I believe that my friends will never betray me 0.792	I look at the bright side of life 0.738	I like to make sure there are no mistakes 0.542	I have difficulty imagining things* 0.795	I sometimes find a solution other people do not see 0.455
I finish what I start 0.734	I like hearing about other cultures and religions 0.779	I am helpful and unselfish with others 0.689	I am relaxed and handle stress well 0.693	I enjoy leading others 0.873	I trust others 0.780	I wake up happy almost every day 0.688	I love learning new things in school 0.532	I find it difficult to create new things* 0.776	I sometimes behave irresponsibly* −0.440
I forget to do what I was asked to do* 0.655	I ask questions about other cultures 0.729	It is important to me that my friends are okay 0.688	I get nervous easily* 0.691	I want to be in charge 0.856	I believe that most people are honest 0.737	I am a happy person 0.684	I think carefully before doing something 0.531	I have little creativity* 0.628	I am original, I come up with new ideas 0.419
I keep working on a task until it is finished 0.645	I learn a lot from people with different beliefs 0.667	I am always willing to help my classmates 0.635	I often feel nervous* 0.682	I like to be a leader in my class 0.813	I believe that my friends can keep my secrets 0.686	I am always positive about the future 0.658	I stop to think before acting 0.517	I like to ask questions 0.369	I like to create things 0.410
I stop when work becomes too difficult* 0.624	I am not interested in other countries and cultures* 0.638	I treat others with respect 0.564	I know how to control my anger 0.668	I dislike leading a team* 0.809	I think most of my classmates can keep their promises 0.634	I enjoy life 0.637	I do not like learning* 0.465		I find new ways to do things 0.369
I often forget to do things I promised* 0.604	I am willing to be friends with people from other cultures 0.637	I am ready to help anybody 0.559	I stay calm even in tense situations 0.666	I am dominant, and act as a leader 0.790	I believe most people are kind 0.631	I believe good things will happen to me 0.543	I am eager to learn 0.461		
I give up easily* 0.603	I want to travel to other countries 0.601	I like to help others 0.532	I often feel angry* 0.626	I am a leader 0.530	I distrust people* 0.603	I am outgoing and social 0.477	I say the first thing that comes to my mind 0.460		
I hate leaving tasks unfinished 0.581	I feel comfortable in new cultural environments 0.565	I know how to comfort others 0.526	I am not easily upset 0.580	I know how to convince others to do what I want 0.493	I believe that other people will help me 0.577	I have difficulties making friends* 0.443	I like learning new things 0.447		
I make sure that I finish tasks 0.565	I am curious about many different things 0.528	I rarely ask others how they are feeling* 0.488	I panic easily* 0.556			I make friends easily 0.422	I am careful with what I say to others 0.418		
I finish things despite difficulties in the way 0.557	I find science interesting 0.371	I am polite, courteous to others 0.466	I change my mood a lot* 0.479			I expect bad things to happen 0.417	I am often worried about something* −0.398		
I often forget my duties* 0.550		I am warm toward others 0.427	I am afraid of many things* 0.370				I avoid mistakes by working carefully 0.387		
I am a responsible person 0.492		I am reliable and can always be counted on 0.695	I can control my actions 0.365				I worry about many things* −0.361		
I avoid responsibilities* 0.407			I get scared easily* 0.351						

Rotation Method: Promax with Kaiser Normalization.

Components: 1. Perseverance and Responsibility; 2. Curiosity and Tolerance toward diversity; 3. Relations with others; 4. Emotional Control and Emotional Resilience; 5. Assertiveness/Leadership; 6. Trust in others; 7. Social optimism; 8. Care and concern for learning; 9. Creativity – Imagination; 10. Creativity – New solutions.

* Reverse scored items.

Correlations between the final constructs and the overall scale were all significant except for component 10 related to *Creativity* – *New solutions* (*r* = 0.094), ranging between 0.352 (component 8 – *Care and concern for learning*) and 0.692 (component 1 – *Perseverance and Responsibility*). Internal consistency levels, as determined by Cronbach’s alpha, were overall good, ranging between 0.677 and 0.926, except for the *Creativity – New solutions* component (*ɑ* = 0.449).

## Discussion

This paper aimed to validate the Portuguese Child/Youth form of the Survey on Social and Emotional Skills (OECD, 2021) as a reliable, comprehensive self-report measure of a large set of social and emotional skills based on OECD approach to social emotional learning. Particularly, we did so with a large Portuguese sample, testing the measure in diverse community and educational settings, and with a heterogeneous set of participants in a national initiative aimed at supporting the implementation of social and emotional intervention programs – the Gulbenkian Academies for Knowledge. The large sample size and the representativeness of the sample stand as notable strengths in this study.

Internal consistency results were good for most subscales, and excellent for the overall scale, indicating a good internal consistency for this version of the scale. Correlations between each subscale and the overall score were moderate to high (except for the *Assertiveness* and *Resilience/Stress Resistance* subscales), indicating the different dimensions are bound by a common underlying construct related to social and emotional skills.

Descriptive results both at the item-level and subscale level indicate overall high scores at pre-test for most subscales, as well as for the overall scale. Mean scores are similar to those found by OECD in its original study (OECD, 2021), with the subscales *Cooperation* and *Curiosity* scoring the highest, and subscales *Assertiveness* and *Resilience/Stress Resistance* showing the lowest scores.

Results on the sensitivity of the measure to change over time show the SSES can be used to measure the impact of educational and community interventions focused on social and emotional skills for children and youth in a variety of settings in Portugal. The decrease in scores for most subscales found at post-test may relate to a phenomenon well documented in the literature, with participants perceiving themselves as less competent in terms of their social and emotional skills as a result of explicitly discussing them in interventions (Martinsone et al., 2022). As for the effect sizes found in testing differences between pre-test and post-test, several reviews on social and emotional learning have confirmed these programs tend to generate small effects sizes (e.g., Payton et al., 2008; Clarke et al., 2015; Tanner-Smith et al., 2018), leading to discuss the suitability of these commonly used standards (i.e., effects sizes) for attesting the efficacy of these interventions.

Self-report measures report typical behaviors, thoughts, and feelings (OECD, 2021). As Duckworth and colleagues (2015) point out, they are better suited than other types of measures for assessing internal psychological states. Such type of measure also promotes children’s voices as they provide information about themselves (Gedikoglu, 2021).

Preliminary exploratory factor analysis results suggest a 94-items, 10-components structure for the Child/Youth Form of the SSES. Although some factors clearly maintain the structure from the subscales from the original study (e.g., *Assertiveness*, *Trust*), other seem to suggest the combination of two (or more) original subscales as a unified construct (such as *Perseverance* and *Responsibility* in component one), suggesting some shared meaning between how these skills are measured by the SSES. Additionally, some items did not load onto any factor, suggesting they may not share meaning with other items previously organized in the same subscale.

However, suggesting the usage of this 94-tem, 10 component structure is precocious. As previously stated, the SSES was tested in 10 different countries, with different social and cultural contexts, providing a cross-cultural comparability. Research has shown there is consensus regarding the main domains of social and emotional skills, their meaning, and how they translate to daily behavior across different cultures around the world (e.g., Chernyshenko et al., 2018), even though cultural incomparability would also be expected (OECD, 2021). Our results, which differ from the 15 subscales structure from the original SSES – Child/Youth form scale, can be due to cultural norms, values or references that provide different meanings to the same concepts (Jager et al., 2018). In the present study, variability in student characteristics within our sample may be a relevant factor. The original SSES study sample in Portugal was from the municipality of Sintra – a mostly urban, culturally diverse city in the greater Lisbon area –, meaning students were likely to share similar community and school settings. In our study, however, a more diverse national sample was used (e.g., mostly rural versus mostly urban settings; high versus low rates of cultural diversity; diverse school and afterschool experiences), leading to the possibility of greater variability within the data. Although more research is needed on the factor structure of this measure, to provide further insight on the suitability of its structure, these results also shed some light on the fact that SEL interventions should have a culturally responsive approach (Hill, 2019).

Secondly, the larger age gap (and the larger number of participants below and above the ages of 10 and 15) may also impact the data, and the factorial structure found in this study. Indeed, research has shown that age is one of the most relevant individual characteristics to impact social and emotional skills, and that these skills develop at different rates, are understood, and translate into behavior differently for different ages (e.g., Denham et al., 2009). More research is necessary on the factor structure of this measure, in order to provide further insight on the suitability of its structure.

### Limitations and recommendations for future research

Although representing an important step toward the use of the SSES as a practical measure of social and emotional skills for professionals, this work faces its limitations. Firstly, the heterogeneous sample (in terms of children/adolescent and family characteristics) may hinder the study of its psychometric properties, as it adds variability due to participants’ characteristics. For instance, it would be beneficial if analysis were made for separate age groups, since we know these skills develop differently throughout childhood and adolescence (OECD, 2021), and may be understood differently by participants of different ages. Future research should explore the validity of this instrument for different age groups in a more detailed manner than in the present study or in the OECD’s SSES report (OECD, 2021). Additionally, when addressing its applicability to different age groups, one of the instrument’s limitations is its inadequacy for children under 8 years old, both due to literacy constraints and to the conceptual framing of the included skills for children of young age. This is similar to what occurs with other SEL measures, as noted by Martinez-Yarza et al. (2023).

Heterogeneity is also present in the data collection procedures employed by the different Academies. Despite there being a script, and protocol recommendations for the administration of the SSES by the Academies, each team adjusted the data collection process to its context and participants. This inevitable diversity in procedures was necessary, in order to better meet the needs and characteristics of each specific intervention, target population, and implementation team. However, it also accounts for some heterogeneity in who administered the survey (a teacher, a facilitator, older participants responded autonomously at home), the report format (online or paper), or the setting in which it took place (the classroom, at home, during one of the program sessions). This stands as a limitation to the quality of the data and could have an impact on the validation of the measure.

Because no other instrument to measure social and emotional skills was administered in the GAK context with a comparable sample – both in size and in characteristics – to the SSES, no analysis on concurrent or convergent validity were conducted. This stands as an important limitation, and a strong recommendation for future research using the SSES.

Further research is necessary on the SSES – Child/Youth form’s factorial structure, since the solution found in this paper is not clear from a theoretical perspective and does not present a great improvement of the instrument’s psychometric properties when compared to its original structure. The fact that the *Energy* subscale was not included in this validation study also stands as a limitation, since there was no data that allowed us to test the validity of the complete 120-item version of this Child/Youth Form.

Similarly, future research should take into consideration individual differences on how these skills develop, to better understand the effectiveness of its measures. Participants’ sex and age, for instance, are key features for the development of social and emotional skills, since research has found individual differences based on these two variables (OECD, 2021). The same is true for family characteristics, such as mother’s educational level, since it is related to socioeconomic status (e.g.: Aarø et al., 2009) and to the child’s success through life (e.g.: Akram and Pervaiz, 2020). The child’s socioeconomic status is also related to differences in the development of social and emotional skills (OECD, 2021).

It is also necessary to validate SSES’s two other forms – for parents and for teachers, also available in Portuguese – as valuable measures of children’s social and emotional skills when reported by meaningful adult figures in their daily lives. Triangulation of informants, by combining the perspectives of children/youth and others around them, ensures greater rigor, quality, and reliability in evaluating these skills, allowing to form a more detailed picture on social and emotional learning and development (Kankaraš et al., 2019). The same is true for methodological triangulation, suggesting the usage of measures beyond self-report and others-report, such as observational tools or situational judgment tests (e.g., Abrahams et al., 2019; Murano et al., 2021).

## Conclusion

The purpose of this paper was to add evidence on a valuable measure for educational, social and community practitioners for evaluating social and emotional skills in their target audiences, as well as the effectiveness of their SEL interventions. Our results, strengthened by a very large and representative national sample, contribute to prove the utility of this measure for educational and community practitioners to inform and guide their works on social and emotional skills with a varied set of participants, adequately measuring their needs and their strengths. It is particularly useful given the diversity of available instruments under different conceptual frameworks and which focus on a specific skill, or subset of skills. The SSES was developed as a comprehensive measure for a large set of social and emotional skills, anchored in a sound, common theoretical framework.

## Data availability statement

The datasets presented in this article are not readily available because the data pertains to several different institutions, being each institution’s responsibility to grant access. Requests to access the datasets should be directed to pgconhecimento@gulbenkian.pt.

## Ethics statement

Ethical approval was not required for the study involving human samples in accordance with the local legislation and institutional requirements because the paper presents only secondary data analysis of data collected by third parties and was completely anonymized prior to analysis. All third parties guaranteed data anonymity, thereby protecting participant identity and maintaining privacy. Written informed consent for participation in this study was provided by the participants’ legal guardians/next of kin.

## Author contributions

CCa conducted data cleaning, data analysis, and wrote sections of the manuscript. JA contributed to the introduction and discussion sections of the manuscript and conducted a final revision. CB conducted data cleaning, contributed to data analysis and interpretation, and a review of the manuscript. CCo conducted data cleaning and a final revision of the manuscript. All authors contributed to the article and approved the submitted version.

## Funding

CCa was funded by the Portuguese Foundation for Science and Technology (FCT) doctoral grant (UI/BD/154443/2022). CB was funded by the FCT through the CEEC institutional funding (CEECINST/00126/2021).

## Conflict of interest

The authors declare that the research was conducted in the absence of any commercial or financial relationships that could be construed as a potential conflict of interest.

## Publisher’s note

All claims expressed in this article are solely those of the authors and do not necessarily represent those of their affiliated organizations, or those of the publisher, the editors and the reviewers. Any product that may be evaluated in this article, or claim that may be made by its manufacturer, is not guaranteed or endorsed by the publisher.

## Footnotes

^1^Scores were obtained by categories related to the Portuguese schooling system: 0 = Cannot read or write; 1 = up to the 4th grade, 2 = up to the 6th grade, 3 = up to the 9th grade, 4 = up to the 12th grade, 5 = university degree.
