# Supplementary material for: Validation of a community-based application of the Portuguese version of the survey on Social and Emotional Skills – Child/Youth Form
Source: Front Psychol. 2023 Aug 21;14:1214032. doi: 10.3389/fpsyg.2023.1214032 (PMC10476092; doi:10.3389/fpsyg.2023.1214032)
Supplement: Supplementary file 1 [file Table_1.docx]

Supplementary Material

Validation of a community-based application of the Portuguese version of the Survey on Social and Emotional Skills - Child/Youth Form

**Catarina Castro, Maria Clara Barata, Joana Alexandre, Carla Colaço**

*** Correspondence:** Catarina Castro: cacco11@iscte-iul.pt

# Supplementary Tables

Supplementary Table 1. Descriptive statistics for the SSES - Child/Youth form items, subscales and overall score at pre-test.

|  | N | Mean | SD | Min | Max | Skewness | Kurtosis |
| --- | --- | --- | --- | --- | --- | --- | --- |
| **Curiosity** | | | | | | | |
| Subscale | 2420 | 4.10 | 0.59 | 1.14 | 5.00 | -0.729 | 0.843 |
| Item 1 | 2415 | 4.14 | 0.88 | 1 | 5 | -1.004 | 0.872 |
| Item 2 | 2409 | 3.96 | 0.91 | 1 | 5 | -0.768 | 0.526 |
| Item 3 | 2405 | 3.92 | 0.99 | 1 | 5 | -0.831 | 0.322 |
| Item 4 | 2410 | 4.19 | 0.84 | 1 | 5 | -1.085 | 1.295 |
| Item 5 | 2407 | 4.33 | 0.77 | 1 | 5 | -1.231 | 2.051 |
| Item 6 | 1743 | 4.09 | 1.10 | 1 | 5 | -1.320 | 1.132 |
| Item 7 | 2411 | 4.11 | 0.87 | 1 | 5 | -1.011 | 1.227 |
| Item 8 | 2401 | 4.06 | 1.01 | 1 | 5 | -1.044 | 0.732 |
| **Responsibility** | | | | | | | |
| Subscale | 2934 | 3.34 | 0.97 | 1.00 | 5.00 | -0.413 | -0.463 |
| Item 1 | 2258 | 3.00 | 1.20 | 1 | 5 | -0.058 | -0.930 |
| Item 2 | 2919 | 3.56 | 1.50 | 1 | 5 | -0.661 | -1.061 |
| Item 3 | 2257 | 3.04 | 1.29 | 1 | 5 | -0.092 | -1.103 |
| Item 4 | 2260 | 3.03 | 1.36 | 1 | 5 | -0.092 | -1.197 |
| Item 5 | 2914 | 3.45 | 1.36 | 1 | 5 | -0.572 | -0.901 |
| Item 6 | 2916 | 3.27 | 1.29 | 1 | 5 | -0.370 | -0.936 |
| Item 7 | 2252 | 3.17 | 1.29 | 1 | 5 | -0.259 | -1.025 |
| Item 8 | 2168 | 3.08 | 1.28 | 1 | 5 | -0.174 | -1.043 |
| **Optimism** | | | | | | | |
| Subscale | 2321 | 3.77 | 0.75 | 1.00 | 5.00 | -0.744 | 0.574 |
| Item 1 | 1624 | 3.17 | 1.20 | 1 | 5 | -0.207 | -0.890 |
| Item 2 | 2276 | 3.89 | 1.00 | 1 | 5 | -0.856 | 0.457 |
| Item 3 | 2282 | 3.42 | 1.16 | 1 | 5 | -0.362 | -0.647 |
| Item 4 | 2290 | 3.40 | 1.13 | 1 | 5 | -0.309 | -0.626 |
| Item 5 | 2301 | 4.38 | 0.91 | 1 | 5 | -1.640 | 2.608 |
| Item 6 | 2280 | 3.78 | 1.04 | 1 | 5 | -0.720 | 0.159 |
| Item 7 | 2269 | 4.06 | 0.96 | 1 | 5 | -0.933 | 0.540 |
| Item 8 | 1614 | 3.80 | 1.22 | 1 | 5 | -0.788 | -0.340 |
| **Emotional Control** | | | | | | | |
| Subscale | 3116 | 3.05 | 0.78 | 1.00 | 5.00 | -0.172 | -0.273 |
| Item 1 | 3088 | 2.98 | 1.26 | 1 | 5 | -0.031 | -1.031 |
| Item 2 | 3081 | 3.09 | 1.31 | 1 | 5 | -0.150 | -1.100 |
| Item 3 | 3083 | 3.17 | 1.39 | 1 | 5 | -0.202 | -1.202 |
| Item 4 | 3080 | 3.13 | 1.42 | 0 | 5 | -0.236 | -1.123 |
| Item 5 | 3083 | 2.93 | 1.30 | 1 | 5 | 0.068 | -1.071 |
| Item 6 | 3072 | 2.98 | 1.29 | 1 | 5 | 0.026 | -1.016 |
| Item 7 | 3085 | 2.88 | 1.29 | 1 | 5 | 0.083 | -1.045 |
| Item 8 | 3085 | 3.24 | 1.39 | 1 | 5 | -0.282 | -1.175 |
| **Self-control** | | | | | | | |
| Subscale | 3123 | 3.24 | 0.92 | 1.00 | 5.00 | -0.428 | -0.538 |
| Item 1 | 3093 | 3.35 | 1.33 | 1 | 5 | -0.480 | -0.927 |
| Item 2 | 3092 | 3.20 | 1.30 | 1 | 5 | -0.326 | -1.002 |
| Item 3 | 3088 | 3.20 | 1.24 | 1 | 5 | -0.298 | -0.870 |
| Item 4 | 3091 | 3.28 | 1.31 | 0 | 5 | -0.438 | -0.742 |
| Item 5 | 3082 | 3.16 | 1.29 | 1 | 5 | -0.197 | -1.004 |
| Item 6 | 3090 | 3.29 | 1.30 | 1 | 5 | -0.384 | -0.940 |
| Item 7 | 3089 | 3.19 | 1.25 | 1 | 5 | -0.295 | -0.887 |
| Item 8 | 3088 | 3.21 | 1.28 | 1 | 5 | -0.250 | -0.967 |
| **Assertiveness** | | | | | | | |
| Subscale | 3105 | 2.67 | 0.89 | 1.00 | 5.00 | 0.257 | -0.458 |
| Item 1 | 3077 | 2.74 | 1.19 | 1 | 5 | 0.157 | -0.796 |
| Item 2 | 3076 | 2.62 | 1.19 | 1 | 5 | 0.292 | -0.735 |
| Item 3 | 3094 | 2.86 | 1.15 | 1 | 5 | 0.026 | -0.706 |
| Item 4 | 3078 | 2.52 | 1.22 | 1 | 5 | 0.386 | -0.764 |
| Item 5 | 2537 | 3.04 | 1.24 | 1 | 5 | -0.013 | -0.900 |
| Item 6 | 3077 | 2.60 | 1.21 | 1 | 5 | 0.355 | -0.739 |
| Item 7 | 3067 | 2.67 | 1.24 | 1 | 5 | 0.263 | -0.860 |
| Item 8 | 3074 | 2.38 | 1.15 | 1 | 5 | 0.504 | -0.520 |
| **Cooperation** | | | | | | | |
| Subscale | 4033 | 4.15 | 0.53 | 1.57 | 5.00 | -0.724 | 1.180 |
| Item 1 | 4009 | 4.42 | 0.73 | 1 | 5 | -1.498 | 3.282 |
| Item 2 | 3990 | 4.14 | 0.79 | 1 | 5 | -0.878 | 1.151 |
| Item 3 | 4013 | 3.95 | 0.89 | 1 | 5 | -0.846 | 0.883 |
| Item 4 | 3473 | 3.80 | 1.04 | 0 | 5 | -0.672 | 0.051 |
| Item 5 | 4020 | 4.31 | 0.72 | 1 | 5 | -1.050 | 1.833 |
| Item 6 | 4018 | 4.19 | 0.83 | 1 | 5 | -1.094 | 1.568 |
| Item 7 | 4006 | 4.22 | 0.79 | 1 | 5 | -1.035 | 1.443 |
| Item 8 | 3995 | 4.14 | 0.80 | 1 | 5 | -0.876 | 1.145 |
| **Sociability** | | | | | | | |
| Subscale | 3457 | 3.82 | 0.68 | 1.00 | 5.00 | -0.587 | 0.176 |
| Item 1 | 3427 | 3.63 | 1.14 | 1 | 5 | -0.593 | -0.378 |
| Item 2 | 3437 | 3.86 | 1.08 | 1 | 5 | -0.739 | -0.202 |
| Item 3 | 3439 | 4.61 | 0.65 | 1 | 5 | -2.031 | 5.431 |
| Item 4 | 2891 | 3.00 | 1.38 | 0 | 5 | -0.084 | -0.959 |
| Item 5 | 3442 | 3.72 | 1.17 | 0 | 5 | -0.894 | 0.545 |
| Item 6 | 3437 | 4.17 | 0.89 | 1 | 5 | -1.064 | 1.078 |
| Item 7 | 3428 | 3.67 | 1.13 | 1 | 5 | -0.638 | -0.322 |
| Item 8 | 2885 | 3.72 | 1.19 | 1 | 5 | -0.716 | -0.361 |
| **Creativity** | | | | | | | |
| Subscale | 2264 | 3.80 | 0.63 | 1.25 | 5.00 | -0.251 | -0.238 |
| Item 1 | 2250 | 4.03 | 0.81 | 1 | 5 | -0.782 | 0.955 |
| Item 2 | 2249 | 3.77 | 0.92 | 1 | 5 | -0.498 | 0.047 |
| Item 3 | 2115 | 3.72 | 1.20 | 1 | 5 | -0.746 | -0.359 |
| Item 4 | 2251 | 3.75 | 0.93 | 1 | 5 | -0.604 | 0.244 |
| Item 5 | 2242 | 3.84 | 1.15 | 1 | 5 | -0.827 | -0.133 |
| Item 6 | 2240 | 4.00 | 0.98 | 1 | 5 | -0.917 | 0.506 |
| Item 7 | 2110 | 3.63 | 1.18 | 1 | 5 | -0.661 | -0.402 |
| Item 8 | 2035 | 3.61 | 1.26 | 1 | 5 | -0.585 | -0.728 |
| **Persistence/Perseverance** | | | | | | | |
| Subscale | 2850 | 3.27 | 1.03 | 1.00 | 5.00 | -0.436 | -0.673 |
| Item 1 | 2829 | 3.27 | 1.34 | 1 | 5 | -0.386 | -1.013 |
| Item 2 | 2820 | 3.32 | 1.33 | 1 | 5 | -0.449 | -0.970 |
| Item 3 | 2822 | 3.34 | 1.41 | 1 | 5 | -0.376 | -1.160 |
| Item 4 | 2823 | 3.26 | 1.32 | 1 | 5 | -0.366 | -1.001 |
| Item 5 | 2819 | 3.31 | 1.32 | 1 | 5 | -0.380 | -0.966 |
| Item 6 | 2821 | 3.21 | 1.30 | 1 | 5 | -0.267 | -1.003 |
| Item 7 | 2809 | 3.26 | 1.37 | 1 | 5 | -0.334 | -1.101 |
| Item 8 | 2824 | 3.22 | 1.30 | 1 | 5 | -0.357 | -0.952 |
| **Resilience/Stress resistance** | | | | | | | |
| Subscale | 1956 | 2.81 | 0.82 | 0.63 | 5.00 | -0.108 | -0.434 |
| Item 1 | 1944 | 3.09 | 1.20 | 1 | 5 | -0.123 | -0.863 |
| Item 2 | 1942 | 2.71 | 1.26 | 1 | 5 | 0.232 | -1.002 |
| Item 3 | 1939 | 1.96 | 1.09 | 0 | 5 | 0.687 | 0.198 |
| Item 4 | 1945 | 2.99 | 1.31 | 0 | 5 | -0.209 | -0.857 |
| Item 5 | 1940 | 3.33 | 1.34 | 0 | 5 | -0.456 | -0.803 |
| Item 6 | 1937 | 2.86 | 1.27 | 1 | 5 | 0.093 | -1.044 |
| Item 7 | 1943 | 2.19 | 0.99 | 1 | 5 | 0.735 | 0.202 |
| Item 8 | 1932 | 3.35 | 1.22 | 1 | 5 | -0.335 | -0.823 |
| **Empathy** | | | | | | | |
| Subscale | 4600 | 3.75 | 0.56 | 1.25 | 5.00 | -0.329 | 0.561 |
| Item 1 | 4568 | 4.06 | 0.83 | 1 | 5 | -0.913 | 1.352 |
| Item 2 | 4579 | 4.57 | 0.69 | 1 | 5 | -2.094 | 6.060 |
| Item 3 | 4572 | 3.62 | 1.02 | 1 | 5 | -0.584 | 0.053 |
| Item 4 | 4562 | 3.73 | 1.03 | 1 | 5 | -0.696 | 0.156 |
| Item 5 | 4553 | 3.29 | 1.02 | 1 | 5 | -0.287 | -0.200 |
| Item 6 | 4555 | 3.63 | 0.93 | 1 | 5 | -0.533 | 0.267 |
| Item 7 | 4528 | 3.67 | 1.01 | 1 | 5 | -0.527 | -0.009 |
| Item 8 | 3907 | 3.36 | 1.26 | 1 | 5 | -0.359 | -0.926 |
| **Tolerance** | | | | | | | |
| Subscale | 3205 | 4.04 | 0.62 | 1.00 | 5.00 | -0.679 | 0.502 |
| Item 1 | 3194 | 4.33 | 0.84 | 1 | 5 | -1.415 | 2.331 |
| Item 2 | 3181 | 3.61 | 1.05 | 1 | 5 | -0.552 | -0.191 |
| Item 3 | 3191 | 3.77 | 1.00 | 1 | 5 | -0.605 | -0.047 |
| Item 4 | 3183 | 4.45 | 0.95 | 1 | 5 | -2.012 | 3.717 |
| Item 5 | 3183 | 4.08 | 0.93 | 1 | 5 | -0.984 | 0.828 |
| Item 6 | 2524 | 4.07 | 1.19 | 1 | 5 | -1.276 | 0.681 |
| Item 7 | 3164 | 3.83 | 0.94 | 1 | 5 | -0.545 | 0.085 |
| Item 8 | 3173 | 4.19 | 0.93 | 1 | 5 | -1.218 | 1.400 |
| **Trust** | | | | | | | |
| Subscale | 3415 | 3.46 | 0.71 | 1.00 | 5.00 | -0.401 | 0.173 |
| Item 1 | 3408 | 3.38 | 0.96 | 1 | 5 | -0.363 | -0.109 |
| Item 2 | 3396 | 3.59 | 1.18 | 0 | 5 | -0.753 | 0.216 |
| Item 3 | 3408 | 3.53 | 1.10 | 1 | 5 | -0.460 | -0.341 |
| Item 4 | 3399 | 3.33 | 1.13 | 0 | 5 | -0.340 | -0.434 |
| Item 5 | 2738 | 3.04 | 1.14 | 1 | 5 | -0.030 | -0.671 |
| Item 6 | 3401 | 3.87 | 0.96 | 1 | 5 | -0.785 | 0.419 |
| Item 7 | 3379 | 3.26 | 1.09 | 1 | 5 | -0.238 | -0.532 |
| Item 8 | 3376 | 3.60 | 1.01 | 1 | 5 | -0.552 | 0.056 |
| Overall score | 6992 | 3.49 | 0.67 | 1 | 5 | -0.881 | 0.971 |
