# Supplementary material for: Validation of a community-based application of the Portuguese version of the survey on Social and Emotional Skills – Child/Youth Form
Source: Front Psychol. 2023 Aug 21;14:1214032. doi: 10.3389/fpsyg.2023.1214032 (PMC10476092; doi:10.3389/fpsyg.2023.1214032)
Supplement: Supplementary file 2 [file Table_2.docx]

Supplementary Material

Validation of a community-based application of the Portuguese version of the Survey on Social and Emotional Skills - Child/Youth Form

**Catarina Castro, Maria Clara Barata, Joana Alexandre, Carla Colaço**

*** Correspondence:** Catarina Castro: cacco11@iscte-iul.pt

# Supplementary Tables

Supplementary Table 2. Detailed factorial structure for the SSES - Child/Youth form.

|  | Components | | | | | | | | | |
| --- | --- | --- | --- | --- | --- | --- | --- | --- | --- | --- |
|  | 1 | 2 | 3 | 4 | 5 | 6 | 7 | 8 | 9 | 10 |
| Persistence – item 5 | **0.848** | 0.007 | -0.025 | -0.085 | -0.048 | 0.020 | -0.010 | 0.047 | -0.034 | -0.096 |
| Persistence – item 4 | **0.734** | -0.136 | 0.062 | -0.195 | -0.031 | 0.018 | 0.159 | 0.057 | -0.034 | 0.042 |
| Responsibility – item 8 | **0.655** | -0.015 | 0.100 | 0.129 | -0.041 | 0.073 | -0.135 | 0.036 | 0.125 | -0.203 |
| Persistence – item 1 | **0.645** | -0.036 | 0.071 | -0.090 | -0.047 | -0.027 | 0.214 | 0.144 | -0.150 | 0.147 |
| Persistence – item 6 | **0.624** | 0.122 | 0.029 | 0.066 | -0.083 | -0.052 | -0.077 | -0.123 | 0.173 | 0.037 |
| Responsibility – item 7 | **0.604** | 0.057 | 0.038 | 0.065 | -0.121 | -0.142 | -0.118 | 0.023 | 0.149 | -0.189 |
| Persistence – item 3 | **0.603** | -0.018 | 0.018 | 0.190 | -0.033 | 0.034 | 0.007 | -0.175 | 0.081 | 0.266 |
| Persistence – item 7 | **0.581** | 0.062 | 0.072 | -0.142 | -0.006 | -0.123 | -0.041 | 0.287 | -0.055 |  |
| Persistence – item 2 | **0.565** | -0.005 | 0.095 | -0.068 | 0.053 | 0.011 | 0.058 | 0.251 | -0.203 | 0.028 |
| Persistence – item 8 | **0.557** | -0.036 | -0.092 | -0.169 | 0.082 | -0.011 | 0.260 | 0.256 | -0.018 | 0.177 |
| Responsibility – item 3 | **0.550** | -0.111 | 0.114 | 0.144 | 0.151 | 0.055 | -0.169 | 0.129 | -0.060 | -0.228 |
| Responsibility – item 6 | **0.492** | -0.067 | 0.242 | -0.022 | 0.089 | -0.023 | 0.088 | 0.255 | -0.177 | -0.172 |
| Responsibility – item 4 | **0.407** | 0.130 | 0.008 | 0.072 | 0.098 | -0.032 | 0.067 | 0.168 |  | -0.348 |
| Tolerance – item 8 | 0.023 | **0.780** | -0.060 | 0.035 | 0.048 | -0.048 | -0.110 | 0.139 | 0.038 | -0.043 |
| Tolerance – item 5 | -0.086 | **0.779** | -0.033 | 0.016 | -0.005 | 0.031 | -0.137 | 0.179 | -0.032 | 0.105 |
| Tolerance – item 2 | -0.044 | **0.729** | -0.029 | 0.068 | 0.022 | 0.048 | -0.002 | 0.098 | -0.047 | 0.249 |
| Tolerance – item 7 | -0.044 | **0.667** | -0.031 | 0.014 | -0.028 | -0.087 | 0.173 | 0.156 | -0.076 | 0.069 |
| Tolerance – item 6 | 0.090 | **0.638** | -0.019 | 0.064 | -0.074 | -0.129 | -0.177 | 0.034 | 0.132 | -0.119 |
| Tolerance – item 1 | -0.063 | **0.637** | 0.250 | -0.053 | -0.105 | 0.013 | -0.011 | -0.031 | -0.084 | 0.022 |
| Tolerance – item 4 | 0.094 | **0.601** | 0.158 | 0.032 | -0.206 | -0.222 | -0.019 | -0.033 | -0.026 | 0.114 |
| Tolerance – item 3 | 0.045 | **0.565** | 0.004 | -0.004 | 0.005 | -0.075 | 0.357 | -0.109 | -0.142 | 0.101 |
| Curiosity – item 1 | -0.082 | **0.528** | 0.218 | -0.012 | -0.020 | -0.025 | -0.096 | 0.061 | 0.148 | 0.264 |
| Curiosity – item 8 | 0.117 | **0.371** | -0.326 | -0.091 | 0.081 | 0.256 | -0.171 | 0.272 | 0.048 | 0.093 |
| Responsibility – item 2 | 0.278 | -0.178 | **0.695** | 0.011 | -0.055 | 0.102 | -0.059 | -0.070 | 0.144 | 0.096 |
| Empathy – item 1 | 0.058 | -0.020 | **0.689** | 0.080 | 0.083 | 0.094 | 0.043 | 0.044 | -0.011 | -0.017 |
| Empathy – item 2 | 0.036 | 0.029 | **0.688** | 0.029 | 0.014 | 0.200 | -0.140 | 0.023 | 0.053 | -0.020 |
| Cooperation – item 6 | 0.006 | -0.025 | **0.635** | -0.009 | -0.021 | 0.246 | 0.002 | 0.032 | 0.027 | 0.021 |
| Cooperation – item 5 | -0.050 | 0.037 | **0.564** | 0.111 | -0.095 | 0.002 | 0.041 | 0.333 | 0.064 | -0.011 |
| Cooperation – item 7 | 0.148 | 0.109 | **0.559** | -0.086 | -0.010 | 0.058 | 0.052 | 0.075 | -0.034 | -0.008 |
| Cooperation – item 1 | 0.100 | 0.239 | **0.532** | 0.052 | -0.023 | 0.012 | 0.001 | -0.027 | -0.093 | -0.022 |
| Empathy – item 4 | 0.145 | -0.048 | **0.526** | -0.041 | 0.303 | 0.021 | -0.124 | -0.084 | 0.044 | 0.209 |
| Empathy – item 8 | 0.067 | -0.098 | **0.488** | -0.081 | 0.106 | 0.049 | -0.147 | 0.019 | 0.377 | -0.280 |
| Cooperation – item 8 | -0.059 | -0.039 | **0.466** | 0.144 | 0.028 | -0.020 | 0.095 | 0.433 | 0.043 |  |
| Empathy – item 7 | -0.079 | 0.140 | **0.427** | -0.003 | 0.032 | 0.004 | 0.121 |  | -0.006 | 0.046 |
| Emotional control – item 3 | -0.088 | 0.111 | 0.227 | **0.761** | -0.090 | 0.018 | -0.132 | 0.119 | -0.023 | -0.170 |
| Resilience – item 1 | -0.260 | -0.095 | 0.068 | **0.693** | 0.092 | -0.130 | 0.242 | 0.272 | 0.055 | 0.192 |
| Resilience – item 2 | -0.082 | -0.091 | -0.009 | **0.691** | 0.102 | -0.071 | 0.222 | 0.014 | 0.062 | 0.117 |
| Resilience – item 6 | -0.045 | -0.076 | -0.051 | **0.682** | 0.097 | -0.018 | 0.192 | -0.076 | 0.208 | 0.080 |
| Emotional control – item 4 | 0.086 | 0.102 | 0.018 | **0.668** | 0.053 | -0.019 | -0.035 | 0.195 | -0.119 | -0.111 |
| Emotional control – item 7 | -0.271 | -0.050 | 0.066 | **0.666** | 0.017 | 0.035 | 0.086 | 0.057 | -0.042 | 0.365 |
| Emotional control – item 8 | 0.199 | 0.019 | 0.064 | **0.626** | 0.039 | -0.011 | -0.021 | 0.073 | 0.064 | -0.271 |
| Emotional control – item 1 | -0.168 | 0.150 | 0.190 | **0.580** | -0.174 | -0.083 | 0.091 | 0.001 | -0.051 | -0.074 |
| Resilience – item 5 | 0.047 | -0.057 | -0.124 | **0.556** | 0.114 | 0.069 | 0.158 |  | 0.062 | 0.101 |
| Emotional control – item 5 | 0.249 | -0.041 | -0.060 | **0.479** | 0.014 | -0.122 | 0.126 | -0.020 | 0.053 | -0.046 |
| Resilience – item 4 | 0.271 | 0.077 | -0.230 | **0.370** | 0.027 | 0.140 | -0.126 | -0.289 | -0.014 | 0.207 |
| Self-control – item 2 | 0.209 | 0.029 | -0.103 | **0.365** | -0.039 | 0.003 | 0.225 | 0.218 | -0.118 | -0.016 |
| Resilience – item 8 | 0.283 | 0.081 | -0.223 | **0.351** | 0.038 | 0.176 | -0.127 | -0.275 | 0.128 | 0.124 |
| Assertiveness – item 7 | -0.047 | -0.007 | 0.064 | 0.064 | **0.916** | -0.053 | -0.017 | 0.053 | -0.126 | 0.043 |
| Assertiveness – item 4 | -0.040 | -0.074 | 0.111 | 0.094 | **0.873** | -0.132 | -0.068 | 0.012 | -0.011 | -0.039 |
| Assertiveness – item 2 | -0.042 | -0.054 | -0.035 | -0.025 | **0.856** | -0.134 | 0.021 | -0.028 | -0.100 | -0.024 |
| Assertiveness – item 6 | 0.024 | 0.138 | -0.088 | -0.011 | **0.813** | 0.084 | 0.013 | 0.008 | -0.088 | 0.049 |
| Assertiveness – item 5 | 0.007 | -0.221 | 0.067 | 0.003 | **0.809** | -0.046 | -0.071 | 0.136 | 0.122 | -0.067 |
| Assertiveness – item 8 | -0.060 | 0.019 | -0.142 | 0.014 | **0.790** | 0.032 | 0.088 | 0.062 | -0.048 | -0.004 |
| Assertiveness – item 1 | 0.156 | 0.105 | -0.052 | -0.040 | **0.530** | 0.021 | 0.237 | -0.008 | 0.052 | -0.001 |
| Assertiveness – item 3 | 0.013 | -0.068 | 0.229 | 0.098 | **0.493** | -0.081 | -0.169 | -0.232 | -0.053 | 0.219 |
| Trust – item 3 | -0.070 | -0.122 | 0.111 | -0.016 | -0.039 | **0.792** | 0.023 | 0.013 | 0.069 | -0.029 |
| Trust – item 8 | -0.008 | 0.013 | 0.097 | -0.067 | 0.030 | **0.780** | 0.098 | -0.044 | -0.109 | 0.033 |
| Trust – item 7 | -0.015 | -0.027 | 0.106 | -0.025 | -0.085 | **0.737** | 0.142 | 0.075 | -0.209 | 0.033 |
| Trust – item 2 | -0.029 | -0.072 | 0.259 | -0.017 | -0.016 | **0.686** | -0.078 | -0.006 | 0.054 | -0.078 |
| Trust – item 1 | 0.071 | -0.145 | 0.098 | 0.008 | -0.145 | **0.634** | 0.071 | -0.039 | -0.023 | 0.202 |
| Trust – item 4 | -0.091 | -0.040 | 0.223 | -0.058 | -0.021 | **0.631** | 0.168 | 0.059 | -0.074 | -0.040 |
| Trust – item 5 | 0.122 | 0.085 | -0.134 | 0.095 | -0.095 | **0.603** | 0.173 | -0.056 | -0.002 | -0.181 |
| Trust – item 6 | -0.129 | 0.051 | 0.099 | -0.101 | -0.067 | **0.577** | 0.254 | 0.190 | -0.099 | -0.111 |
| Optimism – item 6 |  | -0.084 | -0.046 | 0.194 | -0.095 | 0.050 | **0.738** | 0.066 | -0.059 | 0.079 |
| Optimism – item 3 | -0.017 | 0.022 | -0.056 | 0.147 | -0.047 | 0.126 | **0.688** | -0.083 | 0.037 | 0.001 |
| Optimism – item 7 | 0.006 | -0.148 | -0.071 | 0.102 | 0.052 | 0.179 | **0.684** | 0.084 | 0.056 | 0.048 |
| Optimism – item 4 | -0.006 | -0.044 | -0.021 | 0.172 | -0.082 | 0.089 | **0.658** | -0.079 | 0.032 | 0.263 |
| Optimism – item 5 | 0.046 | -0.128 | 0.054 | -0.032 | -0.031 | 0.113 | **0.637** | 0.027 | 0.149 | 0.018 |
| Optimism – item 2 | -0.074 | -0.018 | 0.001 | 0.094 | 0.055 | 0.072 | **0.543** | 0.092 | 0.095 | 0.080 |
| Sociability – item 1 | -0.025 | 0.285 | 0.177 | -0.052 | 0.188 | -0.039 | **0.477** | -0.338 | -0.095 | -0.149 |
| Sociability – item 8 | 0.043 | 0.129 | 0.087 | 0.016 | 0.088 | 0.010 | **0.443** | -0.384 | 0.252 | -0.278 |
| Sociability – item 7 | -0.018 | 0.245 | 0.179 | -0.004 | 0.178 | 0.113 | **0.422** | -0.337 | -0.064 | -0.145 |
| Optimism – item 8 | 0.114 | -0.229 | -0.067 | 0.110 | -0.021 | 0.041 | **0.417** | 0.108 | 0.314 | -0.149 |
| Self-control – item 6 | 0.337 | 0.039 | 0.037 | -0.028 | 0.063 | -0.122 | -0.063 | **0.542** | 0.062 | 0.096 |
| Curiosity – item 7 | 0.065 | 0.203 | -0.061 | -0.115 | 0.112 | 0.223 | 0.071 | **0.532** | 0.113 | -0.030 |
| Self-control – item 3 | 0.051 | -0.001 | 0.136 | 0.442 | -0.042 | -0.012 | 0.042 | **0.531** | -0.042 | 0.021 |
| Self-control – item 7 | 0.098 | 0.157 | -0.092 | 0.370 | 0.023 | 0.058 | -0.095 | **0.517** | -0.167 | -0.038 |
| Curiosity – item 6 | 0.116 | 0.047 | -0.061 | -0.025 | 0.031 | 0.050 | 0.066 | **0.465** | 0.309 | -0.204 |
| Curiosity – item 2 | -0.116 | 0.321 | 0.007 | 0.006 | 0.160 | 0.123 | 0.027 | **0.461** | 0.152 | -0.094 |
| Self-control – item 5 | 0.177 | 0.079 | -0.271 | 0.373 | -0.099 | -0.005 | -0.019 | **0.460** | -0.033 | -0.153 |
| Curiosity – item 5 | -0.016 | 0.272 | 0.099 | -0.085 |  | 0.057 | 0.104 | **0.447** | 0.252 | 0.142 |
| Self-control – item 1 | 0.131 | 0.052 | 0.268 | 0.251 | -0.216 | 0.155 | -0.153 | **0.418** | -0.137 | -0.045 |
| Resilience – item 7 | -0.058 | -0.013 | -0.218 | 0.386 | -0.016 | 0.047 | 0.104 | **-0.398** | -0.008 | -0.008 |
| Self-control – item 4 | 0.275 | 0.078 | 0.127 |  | 0.020 | -0.119 | 0.048 | **0.387** | -0.037 | 0.053 |
| Resilience – item 3 | 0.051 | -0.131 | -0.347 | 0.192 | 0.092 | 0.009 | 0.097 | **-0.361** | -0.023 | -0.048 |
| Creativity – item 3 | -0.030 | 0.062 | 0.050 | -0.008 | -0.161 | -0.098 | 0.137 | -0.032 | **0.795** | 0.123 |
| Creativity – item 7 | -0.122 | -0.031 | 0.104 | 0.154 | -0.130 | -0.118 | 0.148 | 0.050 | **0.776** | 0.159 |
| Creativity – item 8 | 0.127 | 0.013 | -0.075 | -0.029 | 0.135 | 0.038 | -0.045 | 0.103 | **0.628** | 0.102 |
| Curiosity – item 3 | -0.034 | 0.152 | 0.150 | -0.164 | 0.112 | 0.131 | 0.044 | 0.102 | **0.369** | 0.294 |
| Creativity – item 4 | 0.031 | 0.143 | 0.280 | 0.075 | 0.034 | -0.200 | 0.160 | -0.035 | 0.100 | **0.455** |
| Responsibility – item 1 | 0.287 | -0.178 | 0.265 | 0.252 | -0.033 | -0.102 | -0.016 | 0.107 | -0.061 | **-0.440** |
| Creativity – item 2 | 0.006 | 0.198 | 0.006 | -0.089 | -0.057 | -0.083 | 0.288 | 0.088 | 0.262 | **0.419** |
| Creativity – item 5 | -0.234 | 0.246 | 0.044 | 0.263 | 0.162 | 0.226 | -0.325 | 0.029 | 0.166 | **0.410** |
| Creativity – item 1 | 0.112 | 0.162 | 0.050 | 0.058 | -0.063 | -0.120 | 0.285 | 0.103 | 0.171 | **0.369** |
| ɑ | 0.926 | 0.801 | 0.856 | 0.845 | 0.881 | 0.819 | 0.854 | 0.723 | 0.677 | 0.449 |
| Eigenvalue | 17.786 | 8.090 | 5.759 | 4.394 | 3.774 | 2.868 | 2.517 | 2.306 | 2.015 | 1.827 |
| Variance explained | 18.149 | 8.256 | 5.877 | 4.483 | 3.851 | 2.927 | 2.569 | 2.353 | 2.056 | 1.864 |

Rotation Method: Promax with Kaiser Normalization.

Components: 1. Perseverance and Responsibility; 2. Curiosity and Tolerance towards diversity; 3. Relations with others; 4. Emotional Control and Emotional Resilience; 5. Assertiveness/Leadership; 6. Trust in others; 7. Social optimism; 8. Care and concern for learning; 9. Creativity - Imagination; 10. Creativity - New solutions.
